# A Rare Presentation of Pellet Injury in the Neck

**DOI:** 10.5402/2011/306126

**Published:** 2011-04-13

**Authors:** Bulbul Gupta, Achal Gulati, Divya Gupta

**Affiliations:** Department of ENT & Head & Neck Surgery, Maulana Azad Medical College, Jawaharlal Nehru Marg, New Delhi, India

## Abstract

Penetrating neck injuries are dangerous and deserve emergency treatment by virtue of the vital structures present underneath. There is a potential risk of unrecognized vascular injury and retained foreign bodies with their associated complications in these wounds. Therefore, an early diagnostic workup to localize the site of injury and an immediate neck exploration are important.

## 1. Case Report

**Figure 1:**
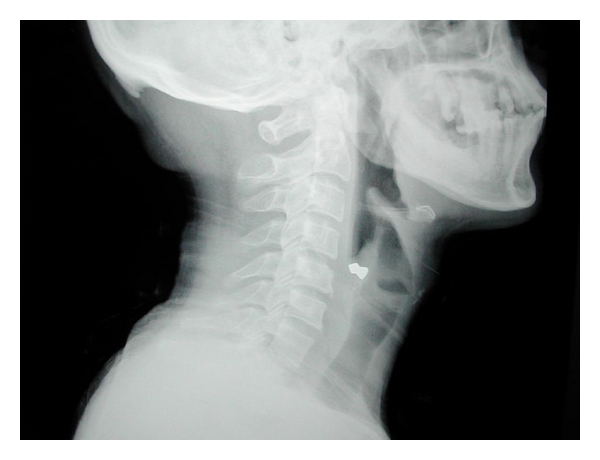
X-ray lateral view showing a radio-opaque foreign body at the junction of C5-C6 vertebrae (level of laryngeal ventricle).

**Figure 2:**
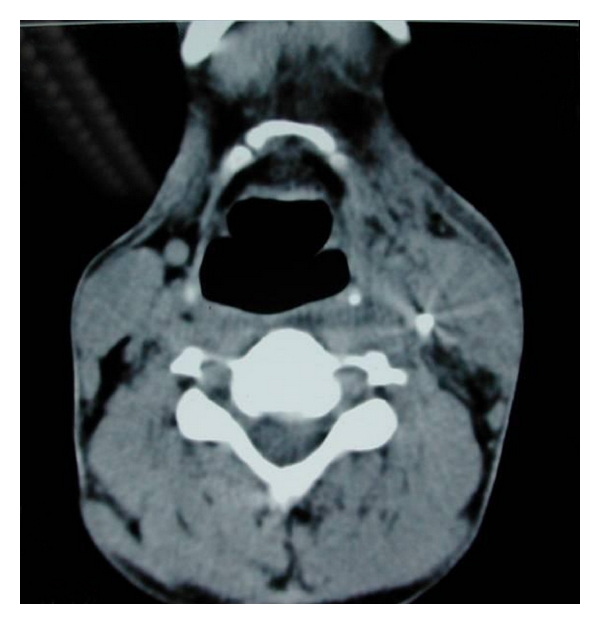
An axial CT scan showing the foreign body deep to the sternomastoid muscle lying in the parapharyngeal space.

**Figure 3:**
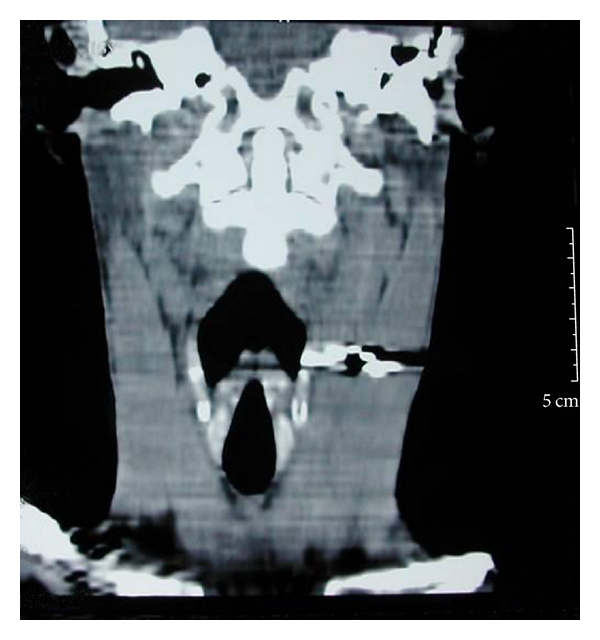
A coronal CT scan of neck showing the radio-opaque foreign body deep in the sternomastoid muscle.

A 45-year-old male presented to the emergency department with alleged history of getting hit over the left side of neck by some air-borne object while standing on the roadside. This was followed up with severe, piercing pain in the neck region associated with bleeding from the wound site. He also gave history of transient loss of consciousness but had no neurological sequelae thereafter. There was no history of difficulty in breathing, change in voice, or swelling over the neck or face. On examination, his vitals were stable. There was no respiratory distress. There was a 2 cm × 1 cm linear lacerated wound on the left side of neck at the junction of upper 1/3rd and lower 2/3rd of the sternomastoid muscle on its anterior border. There was no active bleed or crepitus at the time of examination. No foreign body was visible or palpable in the neck. Indirect laryngoscopic examination was normal. An X-ray of the cervical region showed a radio-opaque foreign body in the left side of neck suggestive of a pellet (Figure 1). A CT scan was ordered before taking up the patient for an exploration and removal of the foreign body. The scan confirmed the position of the pellet as being medial to the sternomastoid around the level of the glottis (Figures 2 and 3).

The neck was explored under general anaesthesia. On going medial to the sternomastoid, a thrill was palpable over the carotid sheath proximal to the foreign body. The pellet was identified within the carotid sheath partially piercing the internal jugular vein such that a part of it was outside the vessel and half of it was within the lumen. The pellet was carefully removed. The bleed was controlled with local pressure, and the vein was ligated and transfixed both proximal and distal to the site of injury.

The postoperative period was uneventful, and the patient is asymptomatic in his follow-up visits.

## 2. Discussion

Airguns have been considered potentially lethal weapons since historical times. They are capable of causing life-threatening injuries. Ktesbias II of Egypt first used compressed air to propel a projectile around 250 BC. Airguns were known as wind chambers, and used an air reservoir connected to a cannon barrel was used. These weapons were used in the Napoleonic wars in the late 17th and early 18th centuries [1]. The modern high-powered rifles can propel a pellet beyond 1100 ft/s (330 m/s), approximately the speed of sound, and produce a noise similar to a  .22-calibre rim-fire rifle. They can generate muzzle velocities of 350 ft/s or more. Petroleum oil placed in the barrel (dieseling) and ignited by the heat produced by the passing pellet results in an explosion which imparts greater velocity and more penetrating power to the pellet. The typical projectile used in rifled airguns is the lead diabolo pellet. This is a wasp-waisted projectile flared at the base, with a variety of head styles. The flared base is designed to improve directional stability [2]. These relatively low-energy missiles produce direct effects on tissues such as laceration and crushing within the missile tract as was also seen in our case. The critical velocity required for the penetration of human skin by an air rifle pellet is around 125–230 ft/s (38–70 m/s), which is well within the muzzle velocities of many air rifles available in the market. A majority of these injuries occur in children and young adolescents. The risk involved with these injuries increases because it is mostly the head and neck region that is affected. There are vital neurovascular bundles, major vessels, trachea, oesophagus, and spinal cord in this region which makes an early diagnosis and immediate management important. Holland et al. have reported three cases of penetrating airgun injuries to the neck [3]: two had the pellet removed and one was managed conservatively. David [4] also published a case involving penetrating injury to the neck in a young adult who had the pellet removed from the posterior oesophageal wall [5].

Penetrating neck injuries can present a difficult diagnostic and therapeutic dilemma. Their evaluation and management remains controversial. Some surgeons advocate mandatory neck exploration while others believe in selective surgical intervention. The universally accepted protocol is that these injuries need to be managed in a systematic manner. First and foremost the airway needs to be established and the cardiocerebral perfusion needs to be maintained. It is after this that a detailed evaluation of the site and severity of the wound needs to be done [6]. Immediate exploration is warranted in the presence of active bleeding, and diagnostic studies should be reserved for those patients who are haemodynamically stable [7]. Injury of major vessels might be tamponaded by foreign bodies; therefore, blind removal of the objects may cause life-threatening hemorrhage. Radiological investigations should be ordered before surgical removal is planned. Preoperative plain radiographs, CT scans, and MRI scans are helpful in giving an idea about the nature and site of injury. They also provide information about any associated complications, forgotten and retained foreign bodies. MRI scans are especially helpful in localising nonmetallic foreign bodies [8]. Radiopaque markers can be used in conventional radiographs in 2 planes as this allows fast, intraoperative localization of radiopaque foreign bodies within soft tissue [5]. Fluoroscopy can also be used intraoperatively to help in localization [9]. Van As et al. [10] have proposed the use of selective angiography in management of gunshot wounds to the neck, along with careful clinical examination particularly so in wounds of the neck and base of skull. Also, if a pellet cannot be seen in the missile tract, there is a possibility of its embolisation to a distant site making angiography important to establish a diagnosis [2]. 

As noted in our case, the plain radiograph and CT scan helped in localizing the pellet and in planning our approach for surgical removal. This prevented excessive bleeding and blind removal of the pellet. Some authors have recommended a no-intervention policy in pellet injuries to the neck. We propose that it should be mandatory to explore all cases with penetrating neck injuries in the light of our patient's findings which suggest that the entry wound might be small enough to make the injury trivial but the missile could be lodged in a vital structure such as the internal jugular vein.
